# All-fiber few-mode interference for complex azimuthal pattern generation

**DOI:** 10.1038/s41598-024-59843-5

**Published:** 2024-04-22

**Authors:** Josué I. Gómez-Méndez, Rodolfo A. Carrillo-Betancourt, Daniel A. May-Arrioja, Amado M. Velázquez-Benítez, Natanael Cuando-Espitia, Juan Hernández-Cordero

**Affiliations:** 1 Applied Physics Group, DICIS, University of Guanajuato, 368850 Salamanca, Guanajuato Mexico; 2 Instituto de Investigaciones en Materiales, UNAM, Cd Universitaria, 04510 Mexico City, Mexico; 3 Centro de Investigaciones en Óptica, Prol. Constitución 607, Fracc. Reserva Loma Bonita, 20200 Aguascalientes, Mexico; 4 Instituto de Ciencias Aplicadas y Tecnología, UNAM, Cd. Universitaria, 04510 Mexico City, Mexico; 5 CONAHCyT, Applied Physics Group, DICIS, University of Guanajuato, 368850 Salamanca, Guanajuato Mexico

**Keywords:** Fibre optics and optical communications, Imaging and sensing

## Abstract

We report on an all-fiber setup capable of generating complex intensity patterns using interference of few guided modes. Comprised by a few-mode fiber (FMF) spliced to a multimodal interference (MMI) fiber device, the setup allows for obtaining different output patterns upon adjusting the phases and intensities of the modes propagating in the FMF. We analyze the output patterns obtained when exciting two family modes in the MMI device using different phase and intensity conditions for the FMF modal base. Using this simple experimental arrangement we are able to produce complex intensity patterns with radial and azimuthal symmetry. Moreover, our results suggest that this approach provides a means to generate beams with orbital angular momentum (OAM).

## Introduction

Structured light with complex intensity patterns can be generated upon adjusting in a controlled manner the phase, amplitude and polarization of an optical wave. These unconventional light beams are of interest for applications ranging from imaging, microscopy, communications, quantum information processing, and light-matter interactions^1–3^. Although a wide diversity of patterns showing distinct interesting features have been demonstrated, light with orbital angular momentum (OAM) is arguably the most familiar example of a structured optical beam^4^. Given the broad scientific and technological impact foreseen for structured light, the conception and realization of alternative tools for creating, manipulating and detecting structured beams is highly relevant and remains as an open challenge in this field for its practical application for different purposes^5,6^. Optical communications for instance, require compact on-chip technologies capable of tailoring and analyzing the quantum features of structured light^1–3,5,6^.

Interference of beams with unconventional modal structure is known to play an important role in the generation of structured light^2^. As an example, the superposition of Laguerre-Gaussian (LG) modes using a Mach-Zehnder interferometer (MZI) has shown to generate higher order mode beams with flower-like structures^7^. Waveguides have also shown to be a suitable platform to fabricate on-chip devices providing adequate modal interaction to generate twisted light^6,8^. Optical fibers in particular provide several means for shaping non-Gaussian optical beams (see^9^ and references therein). All-fiber arrangements typically rely on the modal transformation occurring when the light is coupled from a single-mode fiber (SMF) to fibers supporting higher order modes (e.g., multi-mode or few-mode). Under this approach, the fundamental $LP_{01}$ mode guided by the SMF excites superior radial $LP_{0n}$ modes (i.e., modes of order *n*) in the multi-mode fiber (MMF), and interference amongst these during propagation produces non-Gaussian beams (e.g., Bessel-like, Airy and LG beams)^9^. Because the number of modes supported and propagated along a MMF depend on its core diameter, length, material refractive index and the wavelength, any of these parameters may be adjusted to obtain a specific light pattern at the output^10–12^. The advent of compact all-fiber devices with adjustable modal features thus promises a means to generate light beams with tunable structure.

Photonic lanterns (PLs) mode multiplexers offer an efficient means to produce different modal patterns and have been used to generate beams with OAM^13,14^. Given their modal selectivity, these devices offer increased flexibility for exploring interference effects of degenerate modes (e.g., $LP_{11}^{a}$ and $LP_{11}^{b}$) to produce structured optical beams. Although spatial filtering techniques can provide a means to generate these modes^15,16^, a simpler strategy is operating a SMF at wavelengths below the cut-off, thus exciting higher order modes which can then interfere during propagation. In this approach, the standard SMF acts as a few-mode fiber (FMF) and optical vortex beams with OAM can be generated upon adjusting the phase difference between the $LP_{11}^{a}$ and $LP_{11}^{b}$ modes^17,18^. Similarly, specially tailored optical fibers favoring modal interference have shown to provide a means to generate OAM^19^. Herein we report on the generation of azimuthal intensity patterns obtained through the interaction of the degenerate $LP_{11}^{a,b}$ in a multi-mode interference (MMI) fiber device. In contrast to previous reports in which only the fundamental Gaussian mode is coupled to the MMI device^10–13^, we explore the interference of different modal patterns obtained through adjusting the coupling conditions among the propagating modes in a FMF. Through simulations and experiments, we show that this simple all-fiber arrangement allows for generating complex intensity patterns with both, radial and azimuthal symmetry. Adjustments on the mode coupling conditions lead to changes in the phase and intensity features of the generated patterns, and for some specific cases, beams with features resembling OAM can be readily obtained.

## Experiments and computer simulations

### Experimental setup

**Figure 1:**
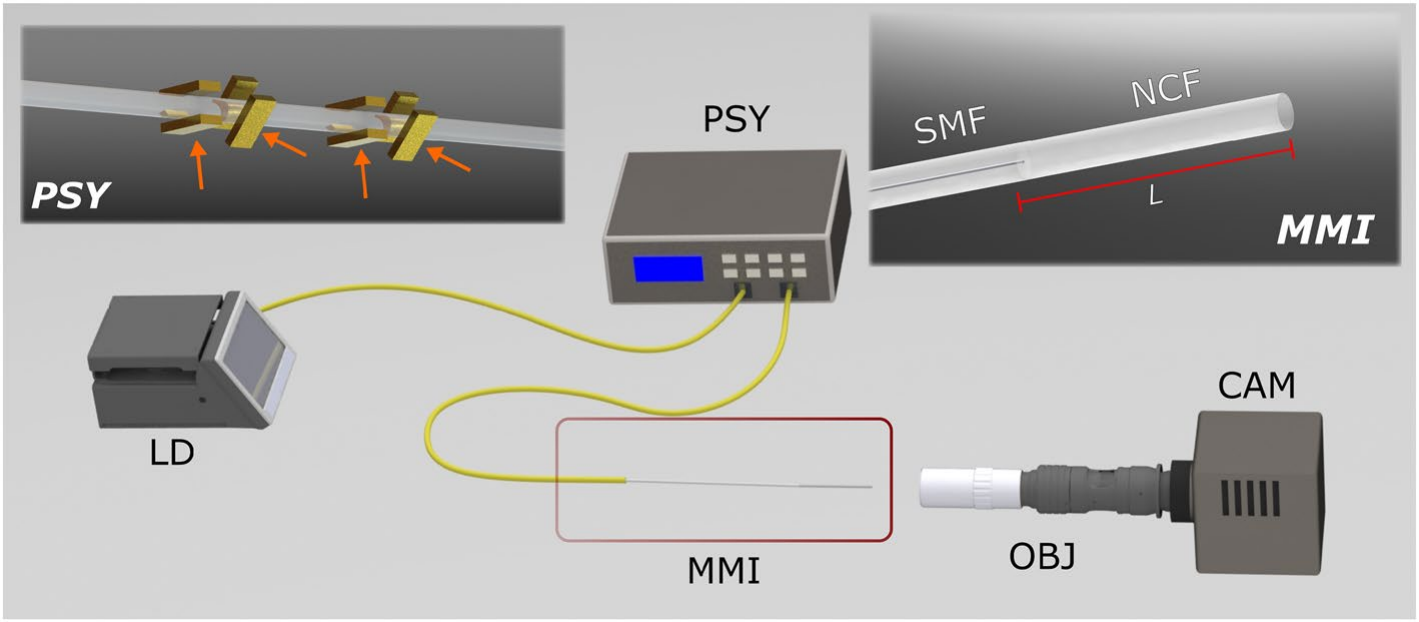
Experimental setup for complex azimuthal pattern generation: a standard SMF-28 fiber (SMF) is used to launch light from a laser diode (LD, 980nm) and it propagates in a few-mode regime. Interference among the excited modes ($LP_{01}$ and $LP_{11}^{a,b}$) is achieved using a multi-mode interference (MMI) device formed upon splicing a segment of no-core fiber (NCF, length=*L*) to the SMF (see inset on the right). The energy distribution and relative phases of the excited modes are adjusted by means of a polarization synthesizer (PSY). A microscope objective (OBJ) and a camera (CAM) are used for recording the patterns at the MMI output end (see text for further details). Polarization and energy distribution of the propagated modes are modified by piezoelectric actuators inside the PSY inducing controlled stress on the fiber indicated in the left side inset by orange arrows.

The experimental setup used to obtain the azimuthal multimodal interference patterns is shown schematically in Fig. 1. A 980nm laser diode (LD, QPhotonics, QFBGLD-980-350) is connected to the input port of a polarization synthesizer/analyzer (PSY 101, General Photonics). The PSY uses a series of in-line piezoelectric fiber squeezers to generate and maintain any state of polarization (SOP) specified by its Stokes parameters, as depicted in the left inset of Fig. 1 (see^20^). Thus, changes on the settings for these parameters can readily modify the SOP of the signal at the output of the PSY. The output of the analyzer is connected to a standard SMF-28 fiber (Corning), and as depicted in the inset of Fig. 1, the MMI devices are simply made upon splicing a segment of no-core fiber of 125$\mu$m diameter (NCF, Thorlabs, FG125LA) at the output end of the SMF. The lengths of the NCF segment in the MMI devices were adjusted using a home-made cleaving station as previously reported^21^. Finally, the setup incorporates an infinity-corrected microscope objective (OBJ) coupled to a CCD camera (Thorlabs, DCC1545M) for recording the output patterns from the MMI. It is worthwhile mentioning that MMIs are bending and temperature sensitive, and therefore care should be taken to control these parameters in order to obtain stable output patterns. For our experiments, the fibers were maintained as straight as possible and the setup was kept at controlled room temperature.

### Initial modal structure

The modal structure of the beam launched at the input of the MMI device was obtained with a laser diode operating at 980nm propagating through a standard SMF-28 fiber. Since the wavelength of the LD is below the cutoff wavelength of the fiber (1250nm), light propagates in a few-mode regime. To determine the number of propagating modes we calculated the normalized frequency (*V*), evaluating first the refractive indices of the core and the cladding at 980nm using Sellmeier’s equation:

1 $$\begin{aligned} n^2(\lambda )=a+ \frac{b\lambda ^2}{\lambda ^2-c} + \frac{d\lambda ^2}{\lambda ^2-e} \end{aligned}$$

In Eq. (1), $\lambda$ is in microns; the coefficients for the core and the cladding used for the calculations were extracted from previous reports^22,23^ and are listed in Table 1. The normalized frequency at $\lambda$=980nm can thus be calculated as $V=3.7$ and accordingly, the supported linearly polarized (*LP*) fiber modes are the fundamental mode ($LP_{01}$) as well as the two degenerate modes $LP_{11}^{a}$ and $LP_{11}^{b}$ (in short, $LP_{11}^{a,b}$). The latter exhibit azimuthal dependence and, as we will show in the next section, they may couple to higher order modes of the MMI device.

**Table 1:** Sellmeier’s coefficients for core and cladding.

Material	a	b	c	d	e
Cladding	1.30956	0.79618	0.01093	0.98716	100
Core	1.31809	0.80709	0.01056	0.98599	100

Because light from the LD propagates in a few-mode regime, the modal content at the output of the FMF will depend on the coupling conditions among the supported modes^24–27^. Experimentally, these were adjusted by means of the PSY, which relies on the controlled birefringence induced by the piezoelectric squeezers located along different positions of an internal fiber segment. Under single-mode propagation, the SOP of the light can be adjusted upon setting the proper Stokes parameters in the PSY. However, in the few-mode regime, the induced birefringence leads also to an energy redistribution of the modal content in the propagating beam via a local modification of the coupling coefficients^27–30^. Hence, the intensity patterns at the output of the FMF can be readily modified upon adjustments in the Stokes parameters.

**Figure 2:**
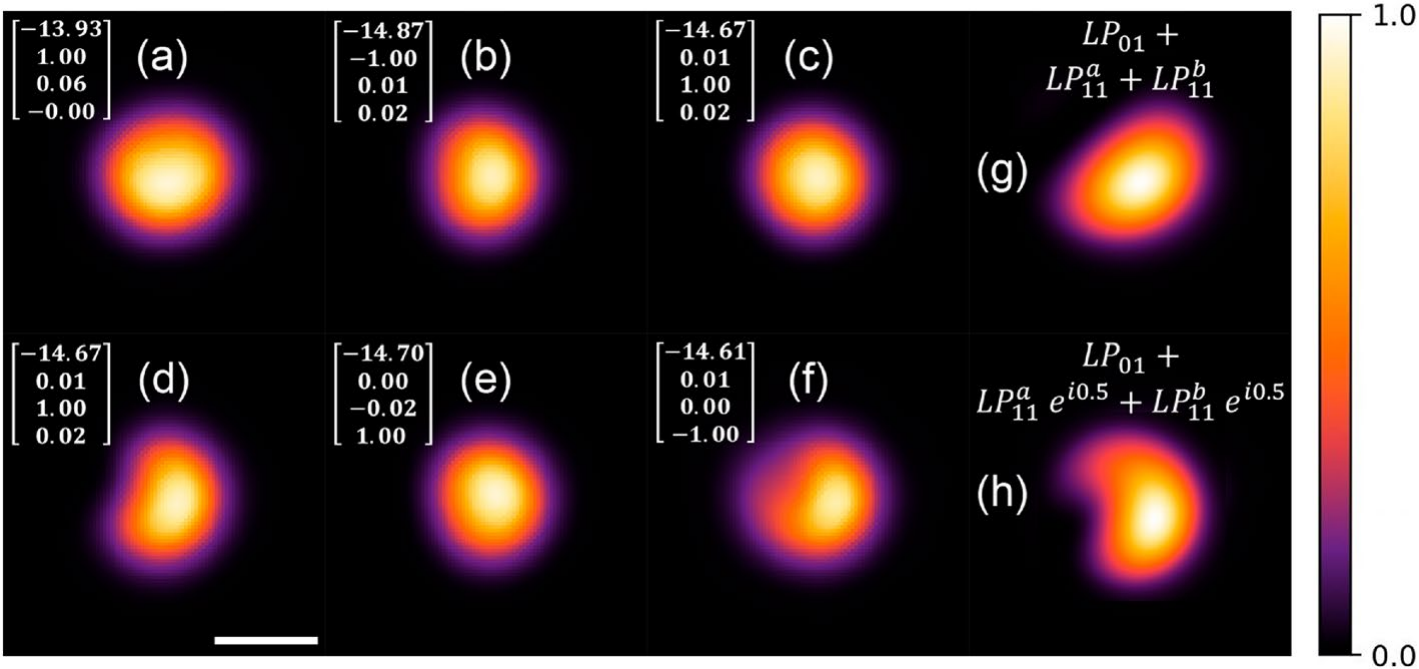
Experimental images of the mode profiles obtained at the output of a standard SMF-28 fiber when launching a 980nm LD. Each pattern corresponds to a SOP defined by the Stokes parameters set in the PSY as shown in the upper left corner: (**a**) linear horizontal polarization, (**b**) linear vertical polarization, (**c,d**) ${\pm }$45^∘^ linear polarization, (**e,f**) right and left circular polarization. The output pattern can be slightly modified through adjustments on the birefringence of the SMF fiber owing to few-mode propagation (see text for details). Simulated intensity patterns with different phase conditions for the propagating modes: (**g**) three modes with the same phase, (**h**) $LP_{11}^{a,b}$ modes with 0.5 radians phase shift with respect to the fundamental mode. The white bar at the bottom left of the figure is 10 $\upmu \textrm{m}$.

Figure 2a–f shows experimental intensity profiles at the output of the FMF for different SOPs set in the PSY (the corresponding Stokes parameters are included in the figure). Notice that changes in the settings yield slightly different intensity patterns owing to the phase differences among the modes resulting from the induced birefringence. In particular, Fig. 2a–f shows the outputs when the PSY is set to yield a linear horizontal polarization, linear vertical polarization, ${\pm }$45^∘^ linear polarization, and right and left circular polarization, respectively. In addition to the experimental observations, Fig. 2g, h includes simulated intensity patterns for two different phase conditions: a null phase shift among the three supported modes with equal amplitude (Fig. 2g), and a phase shift of 0.5 radians between the fundamental mode and the $LP_{11}^{a,b}$ modes (Fig. 2h). Notice that the phase difference indeed yields a change in the calculated output pattern. The PSY has been used previously as a modal controller in the context of Yb-doped fiber amplifiers^31,32^, fiber transmission links at 850 nm^33^, and optical communication encoding^34^. In our experiments, it is a convenient tool to redistribute the modal content launched into the MMI fiber device in a controlled manner. Although an exact modal decomposition for each polarization setting is not attainable with the PSY, we will show in the following sections that small variations in modal content are adequate to generate significantly different intensity patterns at the output of the MMI device.

### Numerical simulations

Calculations of the output intensity patterns from the MMI devices were carried out considering the mode-coupling conditions at the FMF-NCF interface. Coupling into each of the $\nu$ modes of the NCF can be calculated as^10–12^:

2 $$\begin{aligned} \eta _{\nu } = \frac{\int E(\textbf{r}) F_{\nu }(\textbf{r}) \,d\textbf{r}}{\int F_{\nu }(\textbf{r}) F_{\nu }(\textbf{r}) \,d\textbf{r}}. \end{aligned}$$

These excitation coefficients $\eta _{\nu }$ consider the spatial overlap (coordinate $\textbf{r}$) between the field at the end face of the FMF ($E(\textbf{r})$) and the field of each mode of the NCF ($F_{\nu }(\textbf{r})$). In general, $\eta _{\nu }$ quantifies the degree of similarity between the $\nu$ propagating mode and the light entering the NCF. If the optical axes of both fibers are perfectly aligned, the $LP_{01}$ mode of the SMF can only couple to $LP_{0n}$ modes of the FMF, and the excitation coefficients can be denoted as $\eta _{0n}$. Meanwhile, the $LP_{11}^{a,b}$ modes of the SMF couple to the $LP_{1m}^{a,b}$ NCF modes with associated coefficients $\eta _{1m}$. Given the rotational invariance of Eq. (2) and assuming negligible birefringence in the NCF, the excitation coefficients for the degenerate modes are expected to be equal. Under these considerations, the complex field amplitude $U_{out}(\textbf{r})$ at the output end of the MMI ($z=L$) can be expressed as:

3 $$\begin{aligned} U_{out}(\textbf{r})=A\sum _{1}^{n} \eta _{0n} LP_{0n} e^{i(\beta _{0n}L+\alpha )}+G\sum _{1}^{m} \eta _{1m} LP_{1m}^{a} e^{i(\beta _{1m}L+\gamma )}+D\sum _{1}^{m}\eta _{1m} LP_{1m}^{b} e^{i(\beta _{1m}L+\delta )}. \end{aligned}$$

In this expression, the factors $LP_{0n}$ and the $LP_{1m}^{a,b}$ include the spatial distribution of each of the modal families. The propagation constants for each family of modes excited in the NCF are denoted as $\beta _{0n}$ and $\beta _{1m}$, and $\alpha ,\gamma ,\delta$ represent the initial phases of the modes at $z=0$. In addition, we consider a weighted contribution from each mode family through the constants *A*, *G*, and *D*. These constants define the modal content at the end face of the FMF and can be experimentally adjusted by means of the PSY. The pattern at the output of the MMI is thus formed by the superposition of the $LP_{0n}$ and the $LP_{1m}^{a,b}$ modes guided by the NCF, which in turn are excited by the modal superposition at the output of the FMF.

## Results

**Figure 3:**
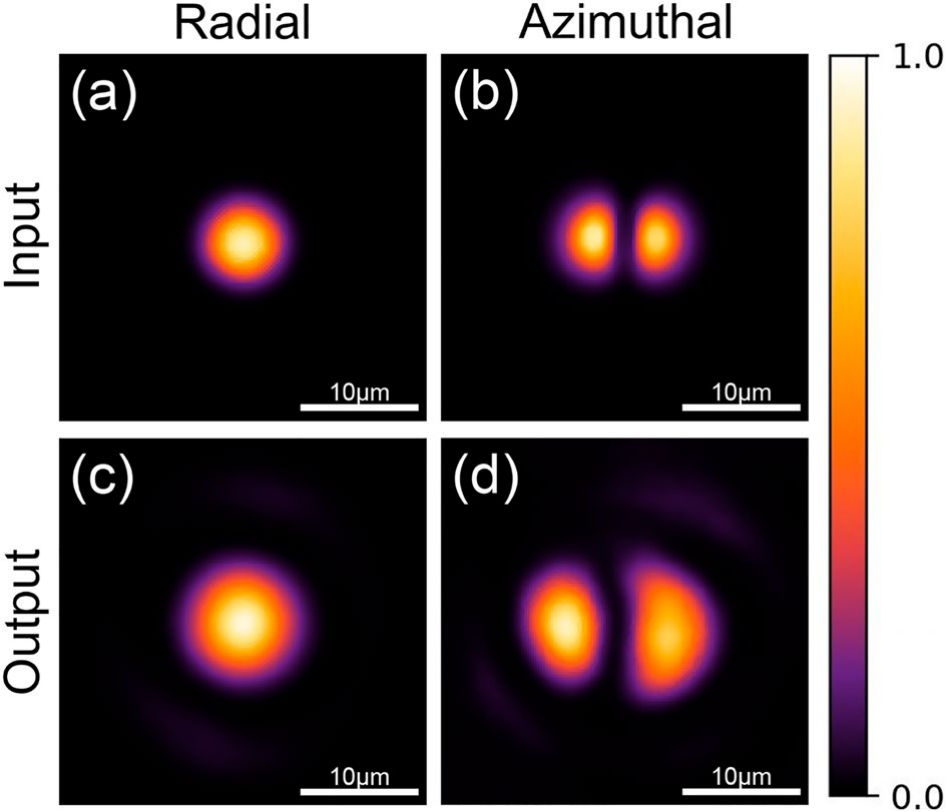
Intensity profiles for the $LP_{01}$ (left) and the $LP_{11}^{a}$ (right) modes obtained at the output of the FMF (upper row) launched into the MMI devices. The self images of these modes are obtained at the output of a MMI device with *L*=93 mm (lower row). See text for further details.

Let us first explore the output of the MMI for each of the three modes used in our experiments. To obtain each mode at the output of the FMF, we used the mandrel wrapping technique and the light from the LD was coupled directly to the MMI. It is known that the length of the NCF in a MMI device plays a critical role on the generated output pattern^10–12^. For instance, the length of NCF required to obtain self-images of the input beam (for a given wavelength, $\lambda$) at the output end face can be estimated as^10^:

4 $$\begin{aligned} L=p\frac{nd^2}{\lambda } \end{aligned}$$

where *n* and *d* are the refractive index and the diameter of the NCF, respectively. The value of *p* defines the number of the self-image; for $p=$4 the output yields almost an exact replica of the field at the FMF-NCF interface because the propagating modes in the MMI are almost in-phase^10–12^. Meanwhile, for $p=$1,2, and 3, we obtain a lossy self-image due to a larger phase mismatch among the propagating modes. Using $p=$4 and for our experimental conditions ($\lambda =$980nm, $n=$1.457 and $d=$125$\mu$m), the self-images of the input modes are expected at *L*=92.9mm. The upper row of Fig. 3 shows the intensity profiles for the $LP_{01}$ and the $LP_{11}^{a}$ modes launched into a MMI device with *L*=93mm (measured within experimental error). As seen in the intensity profile images included in the lower row of the figure, the self-image of each mode can be readily obtained. In other words, the output of the MMI device (lower row images) yields an intensity profile that resembles the initial input field. Notice that these output patterns arise from the ongoing interference of the $LP_{0n}$ modes and the $LP_{1m}$ modes for the radial and azimuthal case, respectively.

**Figure 4:**
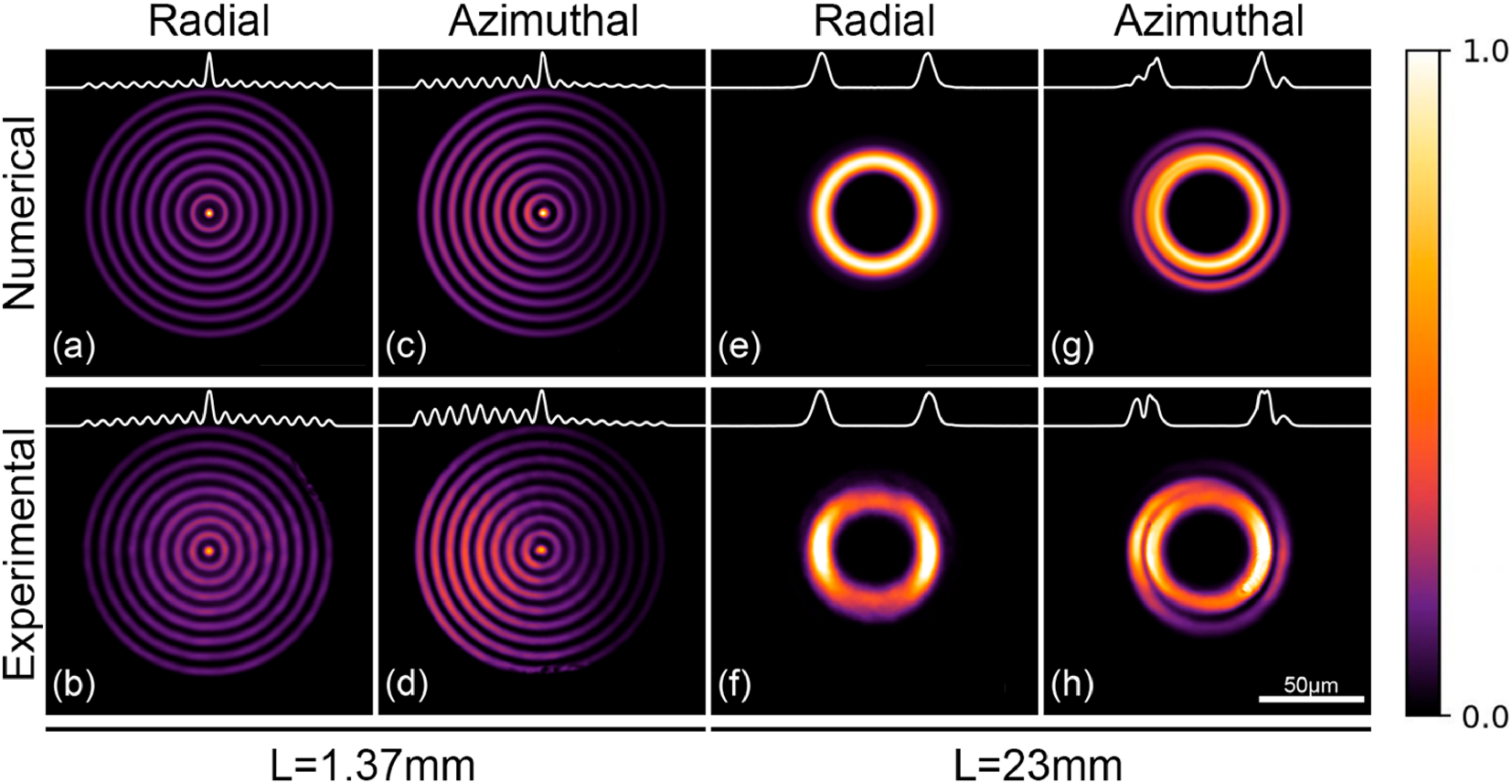
Output patterns from the MMI fiber device obtained upon adjusting the coupling conditions among the modes entering two different lengths (*L*) of the NCF (see text for details). The upper row shows numerical results obtained with Eq. (3) and the lower row includes the intensity patterns obtained experimentally. The white lines at the top of the images are the intensity profiles across the center of each pattern. A series of concentric rings are observed for *L*=1.37mm. The rings in the radial column exhibit constant energy for a given radius (radial symmetry) (**a,b**), while the patterns in the azimuthal column exhibit higher energy on the left side of the images thus breaking the radial symmetry (i.e., they show azimuthal symmetry) (**c,d**). A single annular feature with high radial symmetry is observed for $L=23$ mm. In this case, the azimuthal column shows a similar annular feature with an external and narrow spiral. The columns labeled as radial are obtained launching only the fundamental mode (**e,f**), and those labeled as azimuthal are obtained with a combination of the three modes propagating in the FMF (**g,h**).

We now examine the effects of launching a combination of the three modes into the MMI. Numerically, this was done through adjustments in the coefficients *G* and *D* in Eq. (3), and experimentally, we adjusted the settings of the PSY. Examples of the types of patterns that can be attained are shown in the lower row of Fig. 4, obtained for two representative lengths for the NCF. These two lengths were selected because the outputs resemble intensity patterns observed in beam transformation experiments using MMFs^11,12^. The first length ($L=$1.37 mm) yields a multiple-ring pattern with features such as those observed in *Bessel-like* beams; meanwhile, the second length ($L=$23 mm) provides the first self-image at the output of the MMI device [i.e., $p=$1 in Eq. (4)], showing a ring-like pattern. Let us consider first the case in which only the fundamental $LP_{01}$ mode is launched into the NCF. For the numerical simulations we set $G=D=0$ in Eq. (3) and, in agreement with conventional MMI theory^10^, we obtain light patterns with circular shape, as shown in upper row of Fig. 4 in the columns labeled as “radial”. Upon comparing these two images, the effects of the length of the NCF are evident: while the shortest length (i.e., $L=1.37$ mm) yields multiple concentric rings at the output, a ring-like (or *doughnut-shaped*) pattern is obtained for the larger length ($L=23$ mm). Notice that the intensity output patterns obtained experimentally are closely reproduced by the numerical results obtained with Eq. (3). The MMI therefore transforms the Gaussian beam from the $LP_{01}$ during propagation along the NCF as described in previous reports^11,12^. A similar effect is observed when the $LP_{1m}^{a,b}$ modes are launched into the NCF, although the patterns are no longer purely radially symmetric (see columns labeled as “azimuthal” in Fig. 4). The two patterns (ring-like and concentric rings) are clearly affected by the degenerate modes, as evidenced by the white lines tracing the intensity profiles of the patterns included in the figure. Experimentally, the intensity patterns were modified upon adjusting the SOP settings of the PSY.

**Figure 5:**
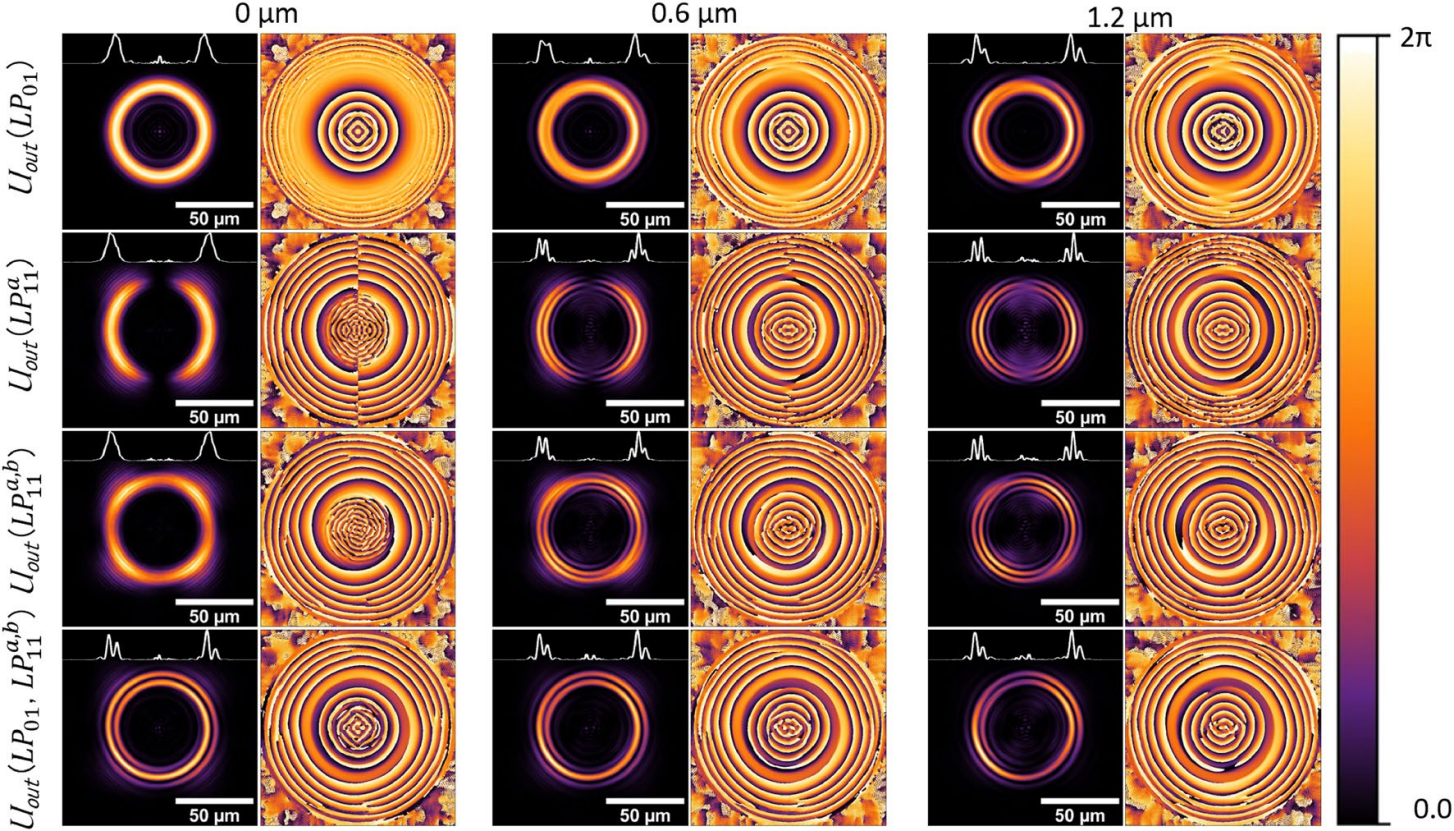
Output patterns and phase maps obtained with BPM simulations (RSoft) for a NCF with $L=23.3$ mm and for different offset conditions (0, 0.6 and 1.2 $\upmu$m) between the optical axes of the FMF-NCF. The first and second rows show the results obtained for a single field distribution at the input of the NCF ($LP_{01}$ and $LP_{11}$ modes, respectively). The patterns included in the last two rows show the results for inputs comprised by a combination of modes with a $\pi$/2 phase difference ($LP_{11}^{a,b}$ third row, $LP_{01}$ and $LP_{11}^{a,b}$ fourth row). The white lines represent the horizontal intensity profiles across the center of each pattern.

Comparing the radial and azimuthal profiles included in Fig. 4, it is evident that launching a combination of modes (i.e., $LP_{01}$ and $LP_{11}^{a,b}$) into the NCF breaks the radial symmetry in the generated patterns. As an example, the pattern with concentric rings obtained for $L=1.37$ mm shows a higher intensity on the left side. Interestingly, the azimuthal profiles for $L=23$ mm show a narrow external spiral-like light distribution around the broader central ring, suggesting that more elaborated patterns can be obtained with further adjustments in the mode phases. In fact, for this particular case (i.e., for $L=23$ mm) the intensity in the central ring obtained experimentally is not perfectly symmetric as one would expect from Eq. (3) for $G=D=0$ (see Fig. 4). We attribute this to a possible misalignment between the optical axes of the SMF and the NCF, a condition that cannot be accounted for with Eq. (3). In order to asses the effect of this offset, we carried out beam propagation method (BPM) simulations using two different programs, OptiBPM and RSoft. Figure 5 includes the output patterns obtained with the BPM for a NCF with $L=23.3$ mm using different modal configurations and offset launching conditions (0, 0.6 and 1.2 $\upmu$m) at the input. It is clear from these images that as the offset between the FMF and the NCF increases, the intensity asymmetries become more evident. Notice that the spiral around the central ring is readily obtained when using the single $LP_{01}$ mode excitation with offset launch (first row), as well as for a combined input including the $LP_{11}^{a,b}$ modes (last row). Off-axis launching of a Gaussian mode has been used previously as a means to excite annular radially polarized modes (i.e., ring-like) supported by a multi-mode fiber^35^. In our case, off-axis excitation arises from the changes in the SOP of the three modes propagating in the FMF. Indeed, adjustments in the PSY not only lead to a phase change among these modes, but also produce a slight off-axis displacement of the field entering the NCF originated from the superposition of the LP modes. The combination of the three modes launched into the NCF thus leads to the propagation and interference of higher order modes, yielding output patterns with more elaborated structures. The supplemental material includes the evolution of the patterns during propagation along the NCF obtained with BPM simulations (OptiBPM). While visualization 1 depicts the patterns obtained when launching each mode independently, visualization 2 shows the patterns obtained when using a combination of modes to excite the NCF.

**Figure 6:**
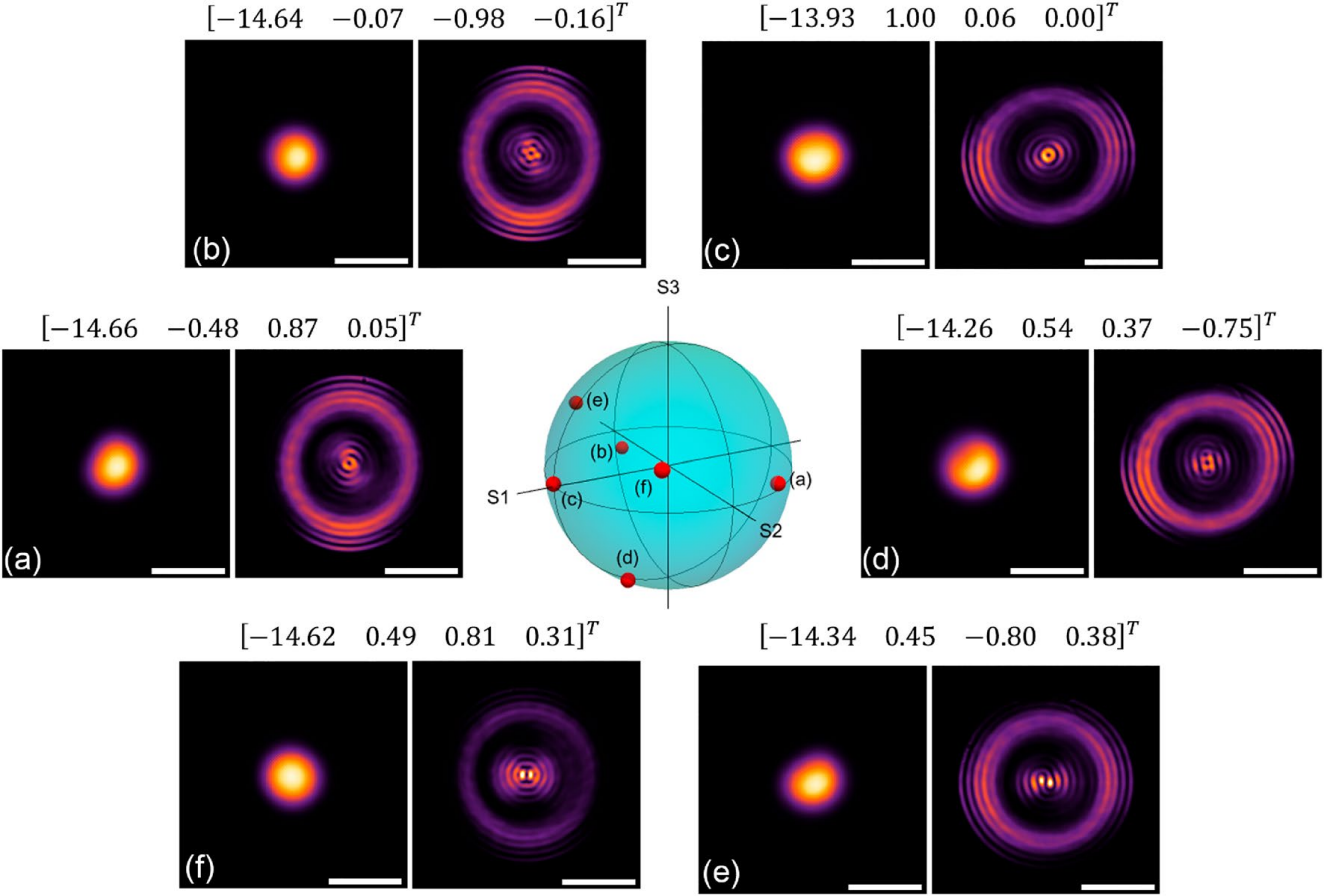
Experimental output patterns obtained under different excitation conditions using a MMI fiber device (NCF length $L=23.7$ mm). Each image was generated upon modifying the SOP in the PSY (the corresponding Stokes parameters are included in the transposed vector on top of each image pair). Each image pair corresponds to an input/output for a given SOP setting. Notice that the main differences among the patterns are within the center region of the fiber tip. The scale bar for the images on the right for each image pair is 50 $\upmu$m, while the scale bar for the images on left is 10 $\upmu$m. Some of these patterns, as well as others, can be obtained through BPM simulations (see visualization 2).

The phase relation among the $LP_{01}$ and the $LP_{11}^{a,b}$ modes entering the NCF affects the intensity patterns obtained at the output of the MMI device. Further evidence of this is shown in Fig. 6, obtained with a MMI device with $L=23.7$ mm. As seen in the figure, changes in the Stokes parameters of the PSY yield different intensity patterns at the output of the FMF. In all cases, the output from the MMI device exhibits an external ring with a diameter close to 80$\mu$m and a central pattern that varies with the settings of the PSY. It is thus clear that the central intensity distribution strongly depends on the modal content at the input of the NCF. Notice that the output patterns are larger than those used as inputs (see the different scale bars in the images), and that seemingly small variations in the input profile generate remarkable differences in the MMI output. In general, the resulting patterns do not resemble the input modal structure, but rather show features that can be attributed to modal interference within the NCF. As an example, the central features of the images shown in Fig. 6a, c exhibit circular symmetry with a small region with minimum intensity exactly at the center of the profile. These images may indicate certain content of orbital angular momentum, or the development of a fractional vortex beam, which has been obtained through interference of Gaussian beams (such as the $LP_{01}$ mode) and the $LP_{11}^{a,b}$ modes^2,9,13,36^. Further evidence of the structured-like features of the resulting patterns can be observed in the third row of Fig. 5, where helical phase fronts are obtained for a combination of $LP_{11}^{a}$ and $LP_{11}^{b}$ modes with phase shift of $\pi /2$ at the input of the MMI. These types of phase fronts are barely noticeable when launching the modes independently and off the center. For some cases, the phase maps are similar to those obtained from the interference of pure $LP_{11}^{a,b}$ modes that are known to yield OAM beams^12,13^. Notice that the output of the MMI yields directly the interference pattern of the modes launched into the NCF. Evidently, the phase relations among the modes and their relative intensities are not fully controllable with the PSY. Nonetheless, the modal interference obtained within the NCF yields complex intensity patterns that can be modified through changes in the SOP set in the PSY.

## Discussion

It is clear that the three modes go through transformations during propagation along the NCF. A single Gaussian mode has been shown to transform into different patterns through multi-mode interference^10–12^. In fact, the *Bessel-like* (concentric rings) and *doughnut-shaped* (ring-like) patterns obtained in our experiments when using only the fundamental $LP_{01}$ mode to excite the NCF, are in agreement with the results reported in^12^. However, launching a combination of modes leads to more elaborated output patterns that are not attainable with the $LP_{01}$ alone. It is also clear that, as demonstrated previously^11,12^, the length of the MMI fiber device plays a critical role on the generated output pattern (see Fig. 4). Evidently, the degenerate $LP_{11}^{a,b}$ modes launched into the NCF will also have an effect in the multimodal interference. This is illustrated in the animation included in visualization 1, which depicts the intensity patterns obtained when the $LP_{01}$ and the $LP_{11}$ modes propagate along different lengths of the NCF. The animation was obtained using OptiBPM from Optiwave, considering the nominal parameters for the SMF and the NCF used in our experiments. Clearly, the length of the NCF will define the type of pattern obtained at the output of the device.

**Figure 7:**
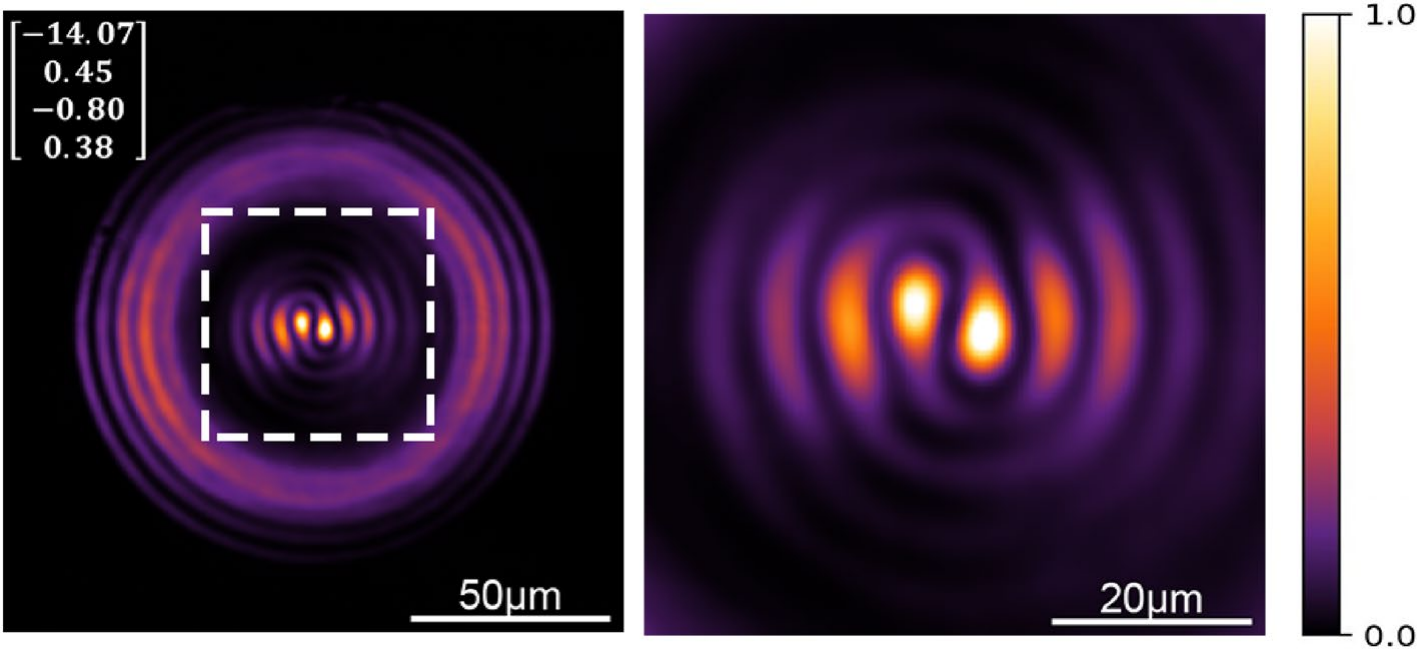
Experimental output pattern from a MMI fiber device using a 23.7 mm long NCF and for a given SOP setting in the PSY (the Stokes parameters are included in the column vector). The image on the right correspond to the region delimited by the dashed line on the left. Some other patterns for this specific NCF length under different PSY settings can be observed in visualization 3.

It has been demonstrated that beams with tunable OAM can be generated upon controlling the polarization in FMFs^17,18^. This is because the phase difference and the coupling conditions between the $LP_{11}^{a,b}$ modes can be readily adjusted with the SOP. Hence, under some stress birefringence conditions in a FMF, the input SOPs (such as those included in Fig. 6) can be mapped to an orbital polarization state at the output^37^. For example, compare the individual patterns of visualization 1 (sec 41) to those shown in Fig. 6. In our experiments, the PSY allows for realizing these adjustments upon setting the appropriate Stokes parameters. Furthermore, offset launching of modes, and phase adjustments within fibers have shown to produce light with OAM^35,38^. All of these effects are readily generated in our devices upon adjusting the SOP in the FMF, yielding intensity patterns that closely resemble the features of structured beams. It is important to note that, when the three modes are launched, the output pattern of the MMI readily yields the interference among all the modes. Hence, for some cases, the intensity patterns will resemble those obtained from the interference between a structured beam and a Gaussian beam; in our setup, the latter is obtained from the fundamental $LP_{01}$ mode, while an OAM beam might result from the combination of the $LP_{11}^{a,b}$ modes. To illustrate this, Fig. 7 includes an output pattern whose central part shows similar features to those obtained from the interference of a beam with OAM of order 2 and a Gaussian beam (see Fig. 5 in^13^). Thus, albeit limited by the purity of the modes generated in the FMF, MMI devices offer versatility to produce a wide variety of complex intensity patterns with a compact all-fiber arrangement (the lengths of NCFs used in our devices were less than 30 mm). The visualizations included in the supplemental material provide an overview of the variety of outputs that would be available using an input with a reconfigurable modal structure (visualization 2 was obtained from simulations and visualization 3 from experiments).

## Conclusions

Multimode interference devices coupled to few-mode fibers provide a promising platform for generating complex intensity patterns. In particular, our results show that the phase relation among the $LP_{01}$ and the $LP_{11}^{a,b}$ modes entering the NCF plays an important role in intensity patterns obtained at the output of the MMI device. Since the output also depends on the length of the NCF and on the wavelength, adjustments on the physical features of the MMIs provide another means to modify the output of these devices. Our results show that the three modes propagating in the FMF can produce different output patterns through adjustments of the SOP using the PSY. Improved control of these patterns might be obtained using mode selective devices such as PLs, allowing for adjusting each individual mode and thus tailor the input field to the NCF. MMI devices may thus provide a simple means to produce structured light upon combining several modes with adjustable phase relations. We should also highlight that the radial dimensions of the generated patterns could be easily scaled by changing the diameter of the NCF, which is a straightforward approach compared to the use specially made waveguides. Additionally, since the response of MMI devices based on NCF can be easily tuned^39^, this could provide a simple way to adjust the patterns using the same length for the NCF. It is important to note that the patterns are obtained in the NCF facet for potential near-field applications. Future work will include the analysis of the free space propagation of such patterns, which could provide even more exciting patterns. An all-fiber device with such capabilities might be of interest for a wide range of applications such as microscopy, microparticle manipulation, and for generation of unconventional light beams such as those with OAM.

### Supplementary Information

Supplementary Legends.

Supplementary Video 1.

Supplementary Video 2.

Supplementary Video 3.

## Data Availability

The datasets used and/or analysed during the current study available from the corresponding author on reasonable request.
