# Supplementary material for: All-fiber few-mode interference for complex azimuthal pattern generation
Source: Sci Rep. 2024 Apr 22;14:9261. doi: 10.1038/s41598-024-59843-5 (PMC11035621; doi:10.1038/s41598-024-59843-5)
Supplement: Supplementary file 1 — Supplementary Legends. [file 41598_2024_59843_MOESM1_ESM.pdf]

All-fiber few-mode interference for complex azimuthal pattern generation.

Supplementary Visualizations captions.

Visualization 1. Visualization of a BPM simulation showing light propagation along a fiber device composed of a SMF-28e, used as FMF, and a no core fiber (NCF) section perfectly aligned in their center. Images illustrate the amplitude distribution of a 975nm wavelength signal. Left side shows the propagation launching the LP_{01} from the FMF into the NCF, whereas the LP_{11}^{a} is launched in the right-side video. The video starts at z=0 and finishes at z=93mm (p=4 in Eq. (4)) as according to MMI theory the intensity patterns replicate for multiples of p=4 in Eq. (4).

Visualization 2. Visualization of a BPM simulation light propagation along a fiber device composed of a SMF-28e, used as FMF, and a no core fiber (NCF) section of 23mm while the LP_{01} and LP_{11}^{a} modes are launched simultaneously in the FMF. Images illustrate the amplitude distribution of a 975nm wavelength signal. Left side shows the case of perfectly aligned FMF and NCF, whereas the right side includes a misalignment of 2 microns.

Visualization 3. Experimental visualization of the intensity patterns generated in the end face of a MMI device comprised by a FMF spliced to a 23.7 mm length NCF. The different patterns are obtained upon modifying the SOP settings in a PSY connected between the light source and the MMI device. The light source used in these experiments was a laser diode operating at 980mn. In particular, the instrument (PSY) uses a triangle wave-based algorithm for scrambling the SOP on the Poincare sphere.
